# Phosphorus Intake and Cancer Risk: A Theoretical–Conceptual Model and Hypothesis for Population-Study Replication

**DOI:** 10.3390/nu18081177

**Published:** 2026-04-08

**Authors:** Ronald B. Brown

**Affiliations:** Waterloo Institute for Complexity and Innovation, University of Waterloo, Waterloo, ON N2L 3G1, Canada; r26brown@uwaterloo.ca

**Keywords:** breast neoplasms/epidemiology, breast neoplasms/etiology, dietary phosphorus, phosphate toxicity, nutritional epidemiology, cancer risk factors, cohort studies, tobacco-cancer studies, conceptual model, confounders

## Abstract

Recent findings in nutritional epidemiology report an association between high dietary phosphorus intake and increased cancer risk. Building on the author’s analysis of breast cancer incidence in the Study of Women’s Health Across the Nation (SWAN), this paper presents a theoretical–conceptual model and a hypothesis to guide further population-study replication. To strengthen the initial SWAN analysis signal, a sensitivity analysis increased the number of controls in the nested case–control design from four to five per case. This adjustment modestly raised the relative risk (RR) of breast cancer incidence among middle-aged women consuming >1800 mg/day of dietary phosphorus (compared with 800–1000 mg/day) from RR: 2.30 to 2.38 (95% CI: 0.95–5.95; *p* = 0.06), improving statistical precision from the original *p* = 0.07. However, the result remains an exploratory pilot signal, not a confirmed association. Because clinical trials cannot ethically expose participants to potential harm from phosphate toxicity, a confirmed association relies on observational research. As in historical tobacco–cancer investigations, secondary analyses are needed across large cohort studies to examine dietary phosphorus intake and incidence of major cancer types. Relevant cohorts include the Nurses’ Health Study, Women’s Health Initiative, Health Professionals Follow-Up Study, National Health and Nutrition Examination Survey (NHANES) Epidemiologic Follow-Up Study, European Prospective Investigation into Cancer and Nutrition (EPIC), and the Canadian Study of Diet, Lifestyle and Health. Effect estimates can be synthesized using meta-analytic methods following PRISMA-P 2015 guidelines. Dietary phosphate modification may offer a cancer prevention strategy with substantial public health impact and clinical implications.

## 1. Introduction

Research in the nutritional epidemiology of cancer has often investigated nutrient deficiencies and the protective effect of vitamins, minerals, and other dietary factors on immune function [1]. Nevertheless, solid tumors evade immune system surveillance [2]. Although several dietary patterns are associated with reduced cancer risk, most nutrients tested in isolation have been found to have minimal protective effects against tumorigenesis [3]. More recently, overload of nutrients and dietary factors such as sodium, fat, sugar, and calories have emerged as determinants associated with chronic disease risk [4]. Among dietary nutrients, overload of the essential mineral phosphorus in the form of dietary phosphate (PO_4_) has not received as much attention.

**Figure 1:**
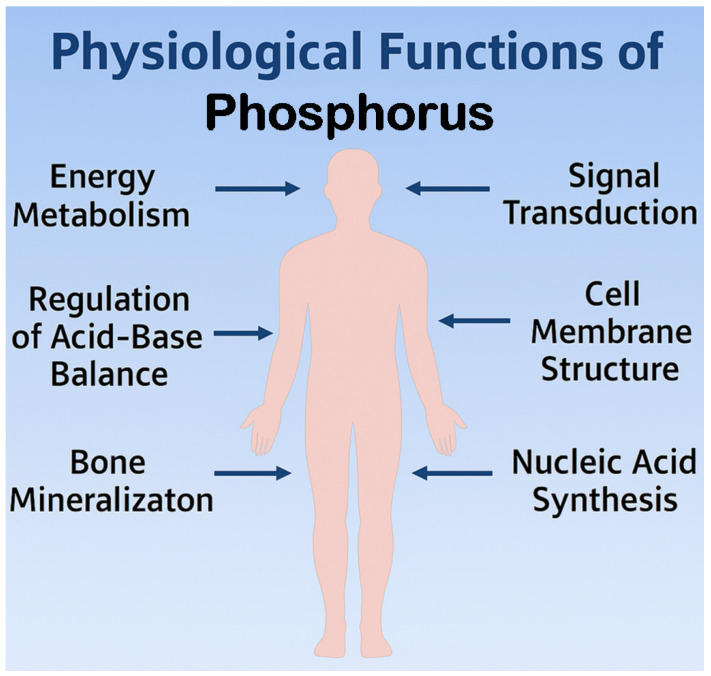
Phosphorus’s physiological functions in the human body.

Phosphorus plays a central role in numerous physiological processes essential to human health (Figure 1) [5]. This mineral plays a central role in energy metabolism as a structural component of ATP, supports the mineralization of bones and teeth, and contributes to the formation of cell membranes through phospholipids. Phosphorus is also required for the synthesis of nucleic acids, DNA and RNA, and participates in the body’s acid–base buffering systems. These functions reflect phosphorus’s importance in maintaining normal cellular activity and overall metabolic health.

Dietary phosphate overload refers to a sustained intake of total dietary phosphate that exceeds the body’s capacity to maintain phosphorus homeostasis [6]. Dietary overload increases intestinal phosphate absorption, thereby increasing the amount of phosphate entering the circulation over time, placing greater regulatory demands on the renal and endocrine systems. Conceptually, excess absorbed dietary phosphate is regulated and redistributed across “phosphorus pools” within the body, such as bone, intracellular compartments, and soft tissues [6]. The result is gradual phosphate accumulation and altered phosphate handling along the continuum from healthy individuals to those with chronic kidney disease. High serum levels of inorganic phosphate (Pi) are associated with phosphate toxicity, the toxic effect of dysregulated phosphate on tissues and organ systems [7].

The hypothesis presented in this paper is grounded in a retrospective synthesis of theoretical concepts established from over a decade of the author’s research in cancer biology and nutritional epidemiology. Findings support the association of excessive dietary phosphate and phosphate toxicity with tumorigenesis through altered cell signaling, increased phosphate transport into cells, angiogenesis, and nucleic acid biosynthesis, which drive dysregulated cellular growth (dysplasia) and proliferation (hyperplasia) [7,8]. Indeed, dietary modification of phosphorus to reduce phosphate overload has been suggested to lower cancer risk [9]. Furthermore, dietary patterns associated with reduced cancer risk are generally lower in phosphorus, including plant-based diets, high-fat ketogenic diets, and low-calorie fasting-mimicking diets [9]. If verified through future population-study replication and meta-analyses, findings of cancer risk associated with phosphate overload and cancer prevention associated with dietary modification have the potential to become one of the next greatest public health achievements [10].

However, careful consideration of methodological precedent and ethical constraints is required to translate theoretical findings of dietary phosphorus overload and cancer risk into public health prevention policies and dietary guidelines. A relevant historical precedent for translating research findings into prevention policy is the association between tobacco use and lung cancer, which was supported by a “confluence of studies from epidemiology, animal experiments, cellular pathology and chemical analytics” [11]. Clinical trials were notably absent from the methodology of this tobacco–cancer model due to potential harm to participants. And yet, tobacco-control research demonstrated that a sustained, population-level intervention of a modifiable exposure could substantially reduce cancer burden.

The epidemiologically based methodology of the tobacco–cancer model is reviewed in this paper and guides the adaptation of similar population-based research supporting an evidence-based phosphorus-cancer model. This paper also reports results of a sensitivity analysis that updates and supports the author’s previous findings of breast cancer incidence associated with high dietary phosphorus, based on a secondary analysis of the Study of Women’s Health Across the Nation (SWAN) [12]. The pilot signal from this small SWAN cohort study can help guide replication and meta-analyses within larger longitudinal cohorts. In parallel, preclinical studies can evaluate phosphorus-related causative pathways through animal feeding experiments and cellular pathology analyses that assess Pi levels within the tumor micro-environment. Another prospect is the possibility of tumor regression and cancer cell reversion [8], which could ultimately motivate clinical testing of dietary phosphate restriction as a neoadjuvant intervention for cancer patients. Modifying phosphorus-related causative pathways in cancer may simultaneously demonstrate both clinical treatment efficacy and population-level prevention. This approach would represent a substantial improvement over the conventional kill/cure paradigm for cancer treatment [13], which is characterized by significant adverse effects and limited long-term efficacy as it targets malignant cells without addressing the underlying metabolic causes of tumorigenesis [9].

The Phosphate Toxicity–Cancer Model presented in this paper is a theoretical–conceptual model [14]. It is a framework that integrates explanatory theory with conceptual mechanisms into a coherent etiology of tumorigenesis. In this framework, the theory proposes general causal processes by which cancers arise from phosphate toxicity, while concepts qualitatively describe the molecular and metabolic mechanisms that support those processes. Molecular mechanisms include endocrine signaling networks that regulate serum inorganic phosphate, and downstream changes in gene expression, protein interactions, and intracellular signaling pathways that govern how cancer cells process information [15]. Metabolic mechanisms support the nutritional epidemiology evidence for dietary phosphate overload, dysregulated serum inorganic phosphate, and tissue Pi sequestration, all associated with angiogenesis and cancer cell biosynthesis and proliferation [16]. The Phosphate Toxicity–Cancer Model integrates these domains by proposing that chronic phosphate overload perturbs metabolic homeostasis and molecular cell signaling in ways that jointly promote tumorigenesis. The advantage of an integrated framework is that it can unify heterogeneous biological observations into a single causal narrative, generate testable predictions, and guide analyses of population-level exposure patterns associated with incident cancer outcomes.

The following hypothesis statement in this paper is predicted by the Phosphate Toxicity–Cancer Model for secondary analyses of large longitudinal cohort studies. Importantly, evidence-based cancer biology pathways underlying the model are not specific to breast tissue and may be generalizable across multiple solid tumor types in analyses of major cohort studies. A suggested path forward recommends beginning with analysis of breast cancer incidence as the primary endpoint in larger cohorts of middle-aged women, to more strictly replicate the controlled conditions of the smaller SWAN study, with the added option to explore other cancer types as secondary endpoints.

**Hypothesis:**  High dietary phosphorus intake is associated with increased breast cancer incidence in middle-aged women, and similar associations may generalize to other major cancer types in both women and men as predicted by the Phosphate Toxicity–Cancer Model.

## 2. Retrospective Synthesis

Over more than a decade, a coherent theoretical model has steadily emerged from research on phosphate toxicity—the accumulation of excess inorganic phosphate due to dietary overload and dysregulated metabolism—as a fundamental determinant of tumorigenesis. Previous work has synthesized evidence that tumor cells express increased phosphate transporters (e.g., sodium phosphate cotransporter 2b [NaPi2b]), store more Pi than normal cells, and depend on elevated intracellular Pi to drive growth-promoting signaling, neovascularization, chromosomal instability, and metastasis [7]. The same work also reviewed how phosphate homeostasis is regulated by the interaction of parathyroid hormone, fibroblast growth factor 23, and bioactive vitamin D to form a bone–kidney–parathyroid–gut axis. The disruption of this axis can lead to phosphate toxicity and widespread tissue injury, including increased risk of cancer and other noncommunicable diseases. The work established phosphate toxicity as a biologically plausible carcinogenic exposure.

### 2.1. Epidemiologic Plausibility and Early Population Signals

Building on this mechanistic and endocrine foundation, epidemiologic studies began to test whether dietary phosphorus overload is associated with cancer incidence in human populations. A nested case–control analysis within the Study of Women’s Health Across the Nation (SWAN) found that women consuming more than 1800 mg/day of dietary phosphorus—levels comparable to those promoted in national dietary guidelines—had an approximately 2.3-fold higher risk of self-reported breast cancer compared with women consuming 800–1000 mg/day, despite limited statistical power [12]. Interpreted through the Bradford Hill criteria, including the observed effect size, dose–response pattern, temporality, specificity, biological plausibility, and coherence with mechanistic evidence, inferred causality was supported and justified for further investigation [12].

### 2.2. Tumor Dynamics, Regression, and Therapeutic Implications

Another paper examined spontaneous tumor regression and reversion, arguing that reducing excessive dietary phosphate may activate these phenomena by depriving tumors of phosphate needed for growth, and noting that ketogenic and fasting-mimicking diets—both low in phosphate—are associated with tumor regression [8]. These insights challenged the kill/cure paradigm and suggested that modifying the phosphate environment could restore more normal cellular behavior.

Finally, several articles translated these mechanistic and epidemiologic insights into concrete therapeutic and public health implications. An interdisciplinary review argued that low-phosphate diets already used in chronic kidney disease management could be repurposed to reduce phosphate levels in the tumor microenvironment, regress tumors, and prevent recurrence, supported by evidence from fasting and ketogenic diets [9]. A broader review of dietary phosphorus in cancer highlighted that typical adult intakes far exceed recommended levels, that low-phosphate diets are compatible with tumor regression, and that national dietary guidelines promote phosphorus-rich patterns associated with increased mortality and breast cancer risk [17]. This work proposed that modulating dietary phosphorus—by reducing intake of high-phosphate grains, protein, and dairy, and increasing intake of fruits and vegetables—could serve as a feasible population-level strategy for cancer prevention.

### 2.3. Synthesis

Taken together, these papers trace a systematic theoretical discovery process in which phosphate toxicity emerges as a central, modifiable determinant of tumorigenesis. The model integrates endocrine regulation, cellular mechanisms, epidemiologic signals, comorbid conditions, tumor dynamics, and dietary patterns into a coherent framework that explains both cancer incidence and progression. The model can be generalized to all cancer types, across biological sexes, and throughout the life course. Further syntheses are needed to construct theories explaining the association of phosphate toxicity with increased cancer risk in menopause and decreased risk with physical activity. This retrospective synthesis provides the conceptual foundation for applying the tobacco–cancer methodological precedent and Bradford Hill’s criteria to the phosphate–cancer model, as well as for designing future population-based, preclinical, and clinical studies to test phosphate-focused interventions.

## 3. Historical Tobacco–Cancer Model

The rationale for including a brief review of the historical tobacco–cancer model in this paper is grounded in the model’s methodological relevance to the phosphate–cancer model. The Bradford Hill criteria were developed in early tobacco research to infer lung cancer causation from observational evidence in lieu of randomized trials. In his 1965 address, *The Environment and Disease: Association or Causation?* [18], Hill illustrated this inferred causative logic with classic examples such as scrotal cancer in chimney sweeps, where a harmful environmental exposure produced a strong, specific, and coherent association long before experimental confirmation of chemical toxicity was possible. The same type of exposure–response pattern that Hill regarded as meaningful in causal inference is reflected in biographical perspectives on phosphorus and cancer. For example, Percy Weston’s *Cancer: Cause & Cure* [19] describes cancer incidence in farm animals following long-term exposure to superphosphate fertilizers and Weston’s own cancer remission after avoiding similar toxic phosphate exposures.

Like in tobacco–cancer research, clinical trials that intentionally expose participants to phosphate toxicity are impermissible [20,21]. Therefore, ethical research on dietary phosphorus and cancer must rely on a similar causal-inference framework. This shared evidentiary structure explains why the Bradford Hill criteria fit the analysis of the SWAN study findings naturally and why the tobacco–cancer model provides an appropriate methodological guide for interpreting observational findings on phosphate overload and cancer risk. Importantly, the reader should always bear in mind that an interpretation of observational evidence based on the Bradford Hill criteria is only an inference, and never a cause in itself.

Although the tobacco–cancer model provides a methodological precedent for causal inference in the absence of randomized trials, the SWAN effect size is no match for the much larger 15- to 30-fold risk of lung cancer associated with decades of tobacco research [22]. Nevertheless, SWAN’s association with breast cancer—even with borderline statistical significance pending larger cohort analyses—substantially exceeds the pooled relative risk of 1.13 in a meta-analysis comparing the postmenopausal stratum of women smokers with never-smokers [23].

Future studies should investigate factors that account for such a wide gap between lung cancer risk and breast cancer risk associated with tobacco smoking, perhaps as a corollary of the Phosphate Toxicity–Cancer Model. For example, phosphate fertilizers are often used excessively in growing tobacco [24], and phosphate additives like diammonium phosphate are used in cigarette manufacturing, with portions transferring into aerosolized constituents in an analysis of mainstream smoke [25]; however, research quantifying inorganic phosphate specifically in tobacco smoke is lacking, representing a potential literature gap. Among over 9500 chemical compounds in tobacco and tobacco smoke, inorganic phosphate is not listed as a specific carcinogenic component by the International Agency for Research on Cancer (IARC) [26]. Hypothetically, direct contact with aerosolized inorganic phosphate breathed into the lungs through tobacco smoke may have a significantly stronger dose–response association with higher lung cancer risk compared to the lower risk of breast cancer associated with lower levels of inorganic phosphate indirectly circulated to the breasts through renal regulation pathways. More research is needed in this area.

Foundational evidence from observational studies that established cigarette smoking as a major cause of lung cancer began in the early 1950s and was provided by two large prospective cohort studies—one in the United Kingdom and one in the United States. In the United Kingdom, Richard Doll and Austin Bradford Hill initiated the British Doctors Study in 1951, enrolling more than 40,000 physicians and following them through repeated questionnaires with linkage to national mortality records. Early findings, published in *BMJ* in 1954, demonstrated a strong, dose-dependent association between cigarette smoking and lung cancer mortality [27]. A subsequent 50-year follow-up report published in 2004 confirmed the long-term mortality effects of cigarette smoking and the substantial benefits of cessation [28]. In the United States, E. Cuyler Hammond and Daniel Horn conducted a prospective cohort study of 187,783 men, reporting markedly elevated lung cancer death rates among smokers in their 1954 *JAMA* publication [29].

These findings underpinned the 1964 U.S. Surgeon General’s Report and subsequent international consensus that tobacco use is a leading preventable cause of cancer [30]. Over the following decades, many high-income countries implemented comprehensive tobacco control policies, including taxation, litigation, advertising bans, smoke-free legislation, health warnings, mass media campaigns, and clinical cessation support [31]. As smoking prevalence declined, lung cancer incidence and mortality in men began to fall, followed later by declines in women as their smoking patterns changed within a lagged timeframe [32]. Women’s smoking uptake occurred later than men’s due to shifting social norms [33] and marketing campaigns aimed at young women, promoting smoking as a symbol of independence and modern womanhood [34].

In very high–Human Development Index (HDI) countries—those with high levels of income, education, and life expectancy—comprehensive tobacco-control policies have already produced sustained declines in lung-cancer mortality [32], whereas in many low- and middle-HDI countries, where tobacco control has been weaker or implemented later, mortality remains low but is projected to rise substantially as the smoking epidemic matures [35]. Modeling studies further quantify the potential impact of aggressive tobacco-control strategies, projecting large gains in quality-adjusted life years, substantial reductions in smoking-related mortality, and significant decreases in health-care costs over multi-decade horizons [36,37].

**Figure 2:**
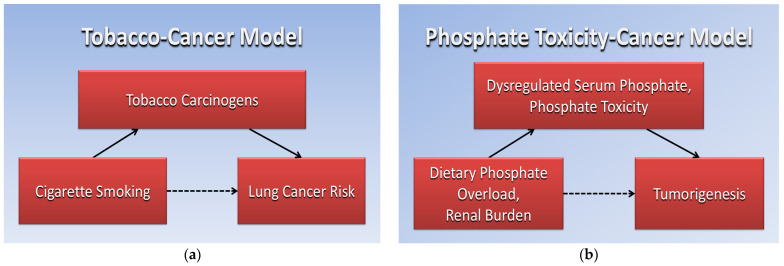
Directed acyclic graphs comparing the Tobacco–Cancer Model (**a**) and the Phosphate Toxicity–Cancer model (**b**). Dotted arrows represent associations and solid arrows represent mediating factors in the causative pathway.

This historical experience is directly analogous to contemporary dietary phosphate modification Just as tobacco control translated observational evidence on smoking and lung cancer into effective, population-level prevention, a coordinated program of observational replication, mechanistic integration, and policy translation could, in principle, do the same for phosphate toxicity and cancer. Figure 2 compares directed acyclic graphs of the Tobacco–Cancer Model (a) and the Phosphate Toxicity–Cancer Model (b). Dotted arrows represent associations and solid arrows represent mediating factors in the causative pathway.

## 4. Sensitivity Analysis of High Dietary Phosphorus and Breast Cancer

The following sensitivity analysis was conducted on the author’s nested case–control study based on data from the Study of Women’s Health Across the Nation (SWAN) [12]. The original analysis of the pilot study found a 2.30 relative risk (RR) of breast cancer incidence in women who consumed the highest dietary phosphorus level of over 1800 mg/day, compared to the reference level of 800–1000 mg phosphorus/day (RR: 2.30, 95% CI: 0.94–5.61, *p* = 0.07).

Phosphorus intake categories were defined a priori in contiguous 200 mg increments from 800 mg to >1800 mg, ensuring uniform spacing across the exposure range. Additionally, most categories correspond directly to established nutritional guidelines or clinical findings in the research literature, including chronic kidney disease dietary recommendations, mortality-associated thresholds, and USDA menu patterns [12]. As these thresholds were determined independently of the SWAN dataset, the analysis does not involve arbitrary subdivision, requiring multiple-testing correction to avoid type I errors. Indeed, the SWAN data show a strong dose–response correlation across these predefined categories, R^2^ of 0.9341 [12]. While the borderline *p*-value reflects limited statistical power in a small cohort, the strong correlation of the category effects supports the interpretation of the SWAN finding as a hypothesis-generating signal that warrants replication in larger datasets.

The more recent sensitivity analysis of the study expanded the number of controls in the primary analysis from four to five per case. Single imputation was applied using SAS 9.4 M8 PROC MI to address missing dietary intake values, and imputed values were calculated as the mean of each participant’s available intake across visits, thereby preserving individual-level dietary patterns.

All methods for handling missing data have limitations, including single imputation (SI), multiple imputation (MI), and last observation carried forward (LOCF). The best method for handling missing dietary data is affected by the type of missingness—random or systematic. LOCF is generally not appropriate for dietary data because nutrient intake can vary from day to day, and carrying a previous day’s intake forward can introduce bias. Importantly, multiple imputation relies on the assumption that missing data are missing at random [38]. However, this assumption is often violated. People tend to skip reporting foods they dislike, forget, or feel uncomfortable reporting, which makes the missingness systematic rather than random. This violation may explain why MI is inaccurate in dietary assessment [39]. By comparison, single imputation does not assume MAR data, and it fills in missing values with a participant’s mean intake rather than carrying the last observation forward. Although not perfect, this justifies SI as a more realistic and defensible choice for handling missing dietary data in the study.

The resulting expanded dataset (see Supplementary Materials) was adjusted for phosphorus caloric density, standardized to a 2000-calorie intake, and categorized into the same phosphorus intake strata as the published primary analysis. Table 1 shows the calculation of relative risks in the sensitivity analysis, which remained directionally consistent with the primary analysis. Relative risk formulas with 95% confidence intervals and *p*-values were calculated to four decimal places using online MedCalc Software Ltd., version 23.4.5 [40]. Statistical significance was set at *p* < 0.05. Along with a slightly higher RR (2.38 compared to 2.30 in the primary analysis), the *p*-value for the highest phosphorus level relative to the reference level improved statistical precision from 0.07 to 0.06 but did not reach the statistical significance threshold of *p* < 0.05. This result represents a pilot signal rather than a confirmed association and should be interpreted as exploratory. Nevertheless, the effect size is the main finding of a quantitative analysis [41], and relative risks greater than 2.0 are unusually large effects for dietary factors associated with cancer risk [42]. Therefore, the SWAN effect size, together with the observed dose–response pattern, mechanistic plausibility, and other causal inferences from the Bradford Hill criteria [12], support a rationale for replication in larger cohorts to further improve statistical significance and confirm an association with high dietary phosphate intake. Importantly, single imputation did not appear to affect results, further underscoring the robustness of the observed association while highlighting the need for replication in larger, independent datasets.

### Rationale for the Adjustment Strategy

The primary analysis adjusted only for total energy intake. This approach is consistent with both the causal structure of the exposure and the design of the nested case–control study. Dietary phosphorus is strongly correlated with total caloric intake; without energy adjustment, the exposure would reflect overall food quantity rather than dietary composition. Energy adjustment, therefore, isolates the nutrient-specific effect of phosphorus and is the standard approach for nutrient–disease analyses.

Additional multivariable adjustment was intentionally avoided to prevent overadjustment bias. Substantial evidence grounded in peer-reviewed findings in the retrospective synthesis of the manuscript suggest that many established breast cancer risk factors (e.g., family history, parity, alcohol use, hormone therapy, menopause, aging, obesity, and smoking) interact with phosphate toxicity, which mediates their associations with tumorigenesis Instead of confounders which lie outside the causative pathway, adjusting for these factors would block part of the causal pathway and attenuate the true effect of phosphorus associated with cancer, leading to biased estimates.

**Figure 3:**
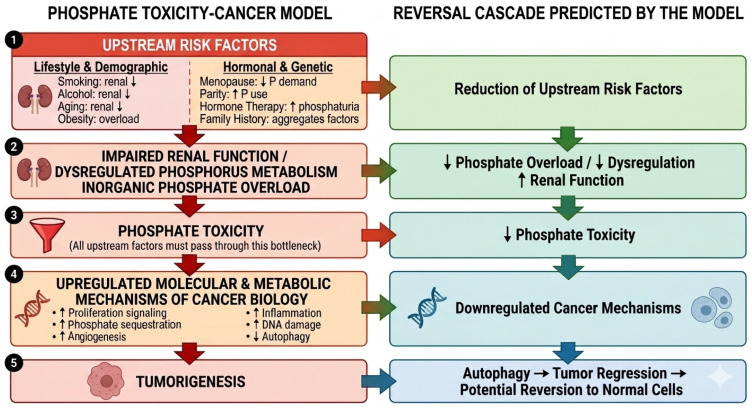
Conceptual cascade of risk factors in the Phosphate Toxicity–Cancer Model. This flow chart shows that upstream risk factors (e.g., smoking, alcohol use, aging, obesity, menopause, parity, hormone therapy, family history) contribute to impaired renal function, dysregulated phosphorus metabolism, and/or inorganic phosphate overload. These processes converge on phosphate toxicity, conceptualized as a bottleneck linking upstream exposures to the molecular and metabolic mechanisms of cancer biology. Phosphate toxicity upregulates pathways that promote tumorigenesis. The model also predicts a reversal cascade: reducing upstream risk factors reduces phosphate toxicity, downregulates cancer-promoting mechanisms, and enables autophagy, tumor regression, and potential reversion of malignant cells toward normal phenotypes.

Figure 3 of the Phosphate Toxicity–Cancer Model conceptualizes phosphate toxicity as a bottleneck that connects directly to cancer biology through molecular and metabolic mechanisms. Consequently, for any variable to function as a risk factor within this model, it must first pass through that phosphate-toxicity bottleneck. In this model, upstream risk factors contribute to impaired renal function, dysregulated phosphorus metabolism, and/or inorganic phosphate overload, which, in turn, produce phosphate toxicity. Phosphate toxicity then upregulates the molecular and metabolic mechanisms of cancer biology, ultimately leading to tumorigenesis.

For example, smoking, alcohol use, and aging are each independently associated with impaired renal function. Recently, a large longitudinal cohort study in Japan demonstrated that cigarette smoking is one of the strongest modifiable predictors of kidney dysfunction in both men and women, accelerating renal decline even after adjustment for other risk factors [43]. Additionally, both acute and chronic alcohol consumption can impair renal regulation of fluid volume and essential electrolytes, specifically disrupting the filtration and excretion of substances such as inorganic phosphate [44]. Aging further exacerbates renal vulnerability: clinical guidelines from the European Menopause and Andropause Society emphasize that kidney diseases are closely linked to the aging process, with age-related decline in renal function impairing mineral homeostasis and predisposing individuals to chronic kidney disease [45]. Together, these exposures reduce renal phosphate clearance and increase susceptibility to dysregulated phosphate metabolism and phosphate toxicity.

Other cancer risk factors, such as obesity, contribute to dietary phosphate overload [17], and the SWAN study suggests that dietary phosphate overload occurs during menopause when phosphate intake remains high while the level of phosphorus needed for reproduction declines dramatically. Inversely, parity may be cancer protective, as increased 1,25-dihydroxyvitamin D released during pregnancy more than doubles intestinal phosphate absorption, demonstrating that markedly increased phosphorus utilization is needed to support reproductive physiology [46]. Furthermore, bazedoxifene, an active ingredient in hormone therapy, increases renal phosphate excretion in postmenopausal women, mitigating dysregulated phosphorus metabolism and improving bone mineral density [47].

Family history may be associated with combinations of these upstream risk factors, and more research is needed in this area. Because the specific variable pathways and mechanisms of risk factors vary by cancer type, a full explanation of each mechanistic corollary of the theoretical–conceptual model would require a separate article and is beyond the scope of the present manuscript. Therefore, a prudent analytic approach during this early exploratory stage is to begin with the basic association using minimal adjustment, and eventually test additional risk factors as model corollaries in future studies.

Importantly, the Phosphate Toxicity–Cancer Model also predicts a reversal cascade: reducing upstream risk factors reduces phosphate toxicity, which downregulates the molecular and metabolic mechanisms of cancer biology, enabling cancer cell autophagy, tumor regression, and potential reversion of malignant cells toward normal phenotypes, all consistently grounded in evidence from the retrospective synthesis [8]. These predictions require empirical verification through future hypothesis-driven studies.

Even adjusting for some types of socioeconomic status (SES) may be inappropriate if SES directly shapes dietary patterns that determine the form of phosphorus exposure, positioning SES as an upstream determinant of mediators lying within the causative pathway rather than an independent confounder. For example, higher-SES women may consume more expensive animal-protein foods containing a high phosphorus-to-protein proportion [48]. In contrast, lower-SES women may consume more inexpensive ultra-processed foods rich in phosphate additives to improve shelf life and flavor [49,50]. The Phosphate Toxicity Cancer Model also predicts that non-dietary SES indicators (e.g., neighborhood deprivation, education, occupation) will not reliably predict breast cancer incidence because they do not directly influence dietary phosphate exposure. This prediction is supported by a 2025 meta-analysis of 17 cohort studies including nearly 800,000 women, which reported no significant association between disadvantaged neighborhood-level SES and breast cancer incidence [51].

Additionally, the SWAN cohort itself was designed to control selection bias by restricting enrollment to middle-aged women undergoing the menopausal transition. Dietary phosphorus intake was assessed using food frequency questionnaires at baseline and other follow-up visits, and none of the participants in the SWAN cohort reported a diagnosis of breast cancer at enrollment. Therefore, dietary assessment preceded all 74 incident breast cancer cases in the nested case–control study, matched to randomly selected controls from the cohort of middle-aged women, establishing temporality of cancer incidence and eliminating the possibility of reverse causality. For these reasons, the energy-adjusted model provides the most causally interpretable estimate of the association between dietary phosphorus and breast cancer risk. In summary, the updated SWAN sensitivity analysis should be interpreted as a pilot signal that warrants replication in larger, independent cohorts. Its borderline-significant association, consistent directionality, and mechanistic plausibility justify a coordinated program of observational replication.

## 5. Recommended Study Replication and Meta-Analyses

The historical trajectory of tobacco–cancer research provides a methodological blueprint for progressively strengthening observational evidence for study replication and meta-analyses when randomized trials are not feasible.

### 5.1. Rationale for a Replication Framework

Early tobacco studies relied on simple measures of association, such as odds ratios from case–control designs that captured differences in disease prevalence between smokers and nonsmokers. As research advances, large prospective cohort studies can be used to estimate relative risks for incident disease. With sufficient follow-up and event timing, hazard-based approaches can quantify how risk accumulates over time. This progression—from prevalence-based associations to incidence-based risks to time-to-event modeling—illustrates how epidemiology strengthens causal inference through increasingly informative observational designs.

### 5.2. Replication in Existing Cohort Studies

A practical next step is to replicate the SWAN pilot signal across large, well-characterized population cohorts that already contain dietary data and cancer outcomes. These include the Nurses’ Health Study, Women’s Health Initiative, Health Professionals Follow-Up Study, National Health and Nutrition Examination Survey (NHANES): Epidemiologic Follow-up Study, European Prospective Investigation into Cancer and Nutrition (EPIC), and the Canadian Study of Diet, Lifestyle and Health. Each cohort offers distinct advantages in sample size, demographic diversity, dietary assessment methods, and follow-up duration.

Applied to the Phosphate Toxicity–Cancer Model, a case–control study can estimate cancer prevalence associated with levels of dietary phosphate, whereas a case–control study nested within a cohort can estimate cancer incidence because cases emerge prospectively during follow-up. Secondary analyses within these datasets can use energy-adjusted models that avoid overadjustment of mediators within the phosphate toxicity pathway. Consistency in the direction, magnitude, and dose–response patterns of effects across cohorts would substantially strengthen the evidence base.

Additionally, the SWAN study provides a valid replication protocol that specifies the analytic details necessary to test the hypothesis that phosphate toxicity is associated with incident breast cancer in large cohorts of middle-aged women. These details include the use of cumulative average dietary assessment, energy adjustment, predefined phosphorus intake categories, and longitudinal follow-up of incident cases [12]. While these parameters offer a clear template, their implementation should be tailored to the structure and measurement characteristics of each cohort dataset.

### 5.3. Meta-Analytic Synthesis

Once multiple cohort analyses are available, effect estimates can be synthesized using meta-analytic methods following PRISMA-P 2015 guidelines [52]. As individual studies report their outcomes in relative terms—typically risk ratios for incident disease in cohort analyses and odds ratios for case–control comparisons—a meta-analysis can pool together either risk ratios or odds ratios to quantify the overall association between high dietary phosphorus and cancer outcomes. Furthermore, heterogeneity analyses can identify whether effect estimates vary across studies and can highlight population subgroups or dietary patterns that modify risk. This approach mirrors the meta-analytic consolidation that ultimately established smoking as a major cause of lung cancer. Importantly, meta-analysis would also allow sensitivity analyses to test the robustness of findings across study designs, confounders, exposure categories, and analytic strategies.

### 5.4. Integration with Mechanistic and Preclinical Evidence

In parallel with population-based replication, preclinical studies can evaluate phosphate-related carcinogenic pathways through animal feeding experiments, tumor microenvironment analyses, and cellular models of phosphate transport and signaling. These mechanistic studies would complement epidemiologic findings by clarifying how phosphate overload influences tumor initiation, progression, and regression. Together, observational replication and mechanistic validation would provide a coherent evidentiary structure for evaluating dietary phosphate as a modifiable cancer risk factor. Over time, preclinical findings could also inform feasibility studies that test dietary phosphate modification in cancer patients.

### 5.5. Advantages of the Replication Roadmap

A major strength of this replication roadmap is its practical accessibility. Many large cohort datasets—including NHANES, EPIC, the Nurses’ Health Study, and the Health Professionals Follow-Up Study—are publicly available or obtainable through straightforward data-use agreements, enabling broad participation in secondary analyses. The analytic methods required to test the phosphate–cancer model rely on simple, transparent measures of association such as risk ratios or odds ratios, rather than complex modeling frameworks. The author’s SWAN study provides an example of a simple analysis for design guidance. With the growing availability of artificial intelligence agents that can assist with data handling, coding, and reproducible workflows, motivated investigators with AI literacy [53]—including trainees and researchers across multiple disciplines—can contribute meaningful replication studies. This accessibility lowers barriers to participation and accelerates the accumulation of evidence needed to evaluate dietary phosphate as a modifiable cancer risk factor.

## 6. Conclusions

The hypothesis proposed in this paper is that high dietary phosphorus intake is associated with increased cancer incidence in middle-aged women, which may be generalized to major cancer types in large longitudinal cohort studies, consistent with predictions of a theoretical–conceptual model. The retrospective synthesis in this paper integrates more than a decade of mechanistic, endocrine, epidemiologic, and theoretical work into the Phosphate Toxicity–Cancer Model, in which phosphate toxicity functions as a modifiable determinant of tumorigenesis. The updated SWAN sensitivity analysis strengthens the initial pilot signal linking high dietary phosphorus intake to increased breast cancer incidence—with borderline statistical significance—while mechanistic evidence supports biological plausibility through altered signaling, increased phosphate transport, and enhanced biosynthesis in dysregulated cells. Because clinical trials cannot ethically expose participants to phosphate toxicity, the methodological precedent of tobacco–cancer research provides a practical framework for advancing the Phosphate Toxicity–Cancer Model through observational replication, mechanistic validation, and meta-analysis synthesis. If replicated across large population cohorts with large and statistically significant relative risks and supported by preclinical studies, these findings could inform dietary guidelines, motivate clinical testing of low-phosphate diets as neoadjuvant interventions, and ultimately contribute to population-level cancer prevention. The potential public health impact is substantial, positioning dietary phosphate modification as a promising strategy for reducing cancer burden and improving long-term health outcomes.

## Figures and Tables

**Table 1:** Sensitivity Analysis with Five Cases Per Control.

Category (mg P)	Cases	Controls	Risk	RR vs. 800–1000	95% CI	*p*-Value
800–1000	6	44	0.120	Reference	—	—
>1000–1200	13	88	0.1287	1.07	0.43–2.65	0.88
>1200–1400	20	104	0.1613	1.34	0.57–3.15	0.50
>1400–1600	14	82	0.1458	1.22	0.50–2.97	0.67
>1600–1800	9	22	0.2903	2.42	0.95–6.14	0.06
>1800	10	25	0.2857	2.38	0.95–5.95	0.06

## Data Availability

https://www.swanstudy.org/swan-research/data-access/ (accessed on 5 December 2025).

## Supplementary Materials

The following supporting information can be downloaded at https://www.mdpi.com/article/10.3390/nu18081177/s1. File S1: Categorized Random Controls_5 Per Case.xlsx.

## Abbreviations

The following abbreviations are used in this manuscript:

ATP	Adenosine Triphosphate
BMD	Bone Mineral Density
BMJ	British Medical Journal
CKD	Chronic Kidney Disease
CKD-MBD	Chronic Kidney Disease-Mineral And Bone Disorder
CI	Confidence Interval
EPIC	European Prospective Investigation into Cancer and Nutrition
FGF23	Fibroblast Growth Factor 23
HDI	Human Development Index
HPFS	Health Professionals Follow-Up Study
IARC	International Agency for Research on Cancer
JAMA	Journal of the American Medical Association
LOCF	Last Observation Carried Forward
MAR	Missing At Random
MI	Multiple Imputation
NHS	Nurses’ Health Study
NHANES	National Health and Nutrition Examination Survey
NSAID	Nonsteroidal Anti-Inflammatory Drug
Pi	Inorganic Phosphate
PRISMA-P	Preferred Reporting Items for Systematic Review and Meta-Analysis Protocols
RNA	Ribonucleic Acid
RR	Relative Risk
SES	Socioeconomic Status
SI	Single Imputation
SWAN	Study of Women’s Health Across the Nation
UVB	Ultraviolet B Radiation
